# Ethanol Extract of Abnormal Savda Munziq, a Herbal Preparation of Traditional Uighur Medicine, Inhibits Caco-2 Cells Proliferation via Cell Cycle Arrest and Apoptosis

**DOI:** 10.1155/2012/926329

**Published:** 2011-07-07

**Authors:** Abdiryim Yusup, Halmurat Upur, Anwar Umar, Benedicte Berke, Nicholas Moore

**Affiliations:** ^1^Faculty of Traditional Uighur Medicine, Xinjiang Medical University, Urumqi 830011, China; ^2^Faculty of Pharmacy, Xinjiang Medical University, Urumqi 830011, China; ^3^Department of Pharmacology, University Victor Segalen Bordeaux 2, 33076 Bordeaux, France

## Abstract

*Aims*. Study the effect of Abnormal Savda Munziq (ASMq) ethanol extract on the proliferation, apoptosis, and correlative gene, expression in colon cancer cells (Caco-2) to elucidate the molecular mechanisms responsible for the anticancer property of Abnormal Savda Munziq. *Materials and Methods*. ASMq ethanol extract was prepared by a professional pharmacist. Caco-2 cells were treated with different concentration of ASMq ethanol extract (0.5–7.5 mg/mL) for different time intervals (48 and 72 h). Antiproliferative effect of ASMq ethanol extract was determined by MTT assay; DNA fragmentation was determined by gel electrophoresis assay; cell cycle analysis was detected by flow cytometer; apoptosis-related gene expression was detected by RT-PCR assay. *Results*. ASMq ethanol extract possesses an inhibition effect on Caco-2 cells proliferation, induction of cell apoptosis, cell cycle arrest in sub-G1 phase, and downregulation of bcl-2 and upregulation of Bax gene expression. *Conclusion*. The anticancer mechanism of ASMq ethanol extract may be involved in antiproliferation, induction of apoptosis, cell cycle arrest, and regulation of apoptosis-related gene expression such as bcl-2 and Bax activity pathway.

## 1. Introduction

Cancer is the second leading cause of death worldwide. Cancer continues to represent the largest cause of mortality in the world. China is confronted with an increasing incidence of cancer and cancer deaths annually. Mortality that results from the common forms of cancer will be unacceptably high in the 21th century. Despite many therapeutic advances in the understanding of the processes in carcinogenesis, overall mortality statistics are unlikely to change until there is a reorientation of the concepts for the use of natural products as new chemopreventive agents [1–4]. 

Natural products include thousands of compounds that exist in fruits, vegetables, plants, and herbs, and several clinical anticancer drugs have been derived from natural products. Thus, development of compounds with anticancer effects from natural products has currently become a very important topic. Natural compounds isolated from medicinal plants, as rich sources of novel anticancer drugs, have been of increasing interest since then [5, 6]. Cancer prevention and treatment using traditional Chinese medicines have also attracted increasing interest, and the development of pharmacology and molecular biology makes it possible to screen effective extracts with anticancer activity from the Traditional Uighur medicinal herbs and many relevant prescriptions. 

Traditional Uighur Medicine, the main part of Traditional Chinese Medicine, has been used for pharmaceutical and dietary therapy for several millennia. Traditional Uighur medicine has its own traditional theory for prevention and treatment of cancer with prescriptions containing Abnormal Savda Munziq (ASMq) [7]. To date, the anticancer effects of ASMq and its potential mechanism of action have been studied on HepG2 cells, Hela cells, T lymphoma cells, and breast cancer cells. Furthermore, the total flavonoids of ASMq were proved to be the most important anticancer-active ingredients of ASMq [8]. In the present study, we used human colon cancer cells (Caco-2) to examine the anticancer effects of ASMq ethanol extract to assess the potential effect of Abnormal Savda Munziq (ASMq) ethanol extract on colon carcinogenesis.

## 2. Materials and Methods

### 2.1. Chemicals

Dulbecco's modified eagle medium (DMEM), RPMI1640, foetal calf serum (FCS), ethylenediamine tetraacetic acid (EDTA), ribonuclease A (RNase A), proteinase K, ethidium bromide, N-Lauroyl sarcosine, propidium iodide, trypsine-0.02% EDTA mixture were obtained from Sigma-Aldrich (Lyon, France). SV Total RNA Isolation System, Reverse Transcription System, dNTPs Mixture, Taq DNA polymerase, BenchTop 1kb DNA Ladder, DNA loading buffer were all from Promega, France. Oligonucleotides used in PCR were from Eurogentec S.A, Britsh. All other chemicals used were of analytical grade. 

### 2.2. Preparation of ASMq Ethanol Extract

ASMq is composed of 10 kinds of herbs [9]. Herbs used in this study were obtained from Xinjiang Hospital of Traditional Uighur Medicine (Urumqi, China). High quality herbs were selected by a specialist, mixed according to the relevant recipe, and the ethanol extract was prepared by a professional pharmacist [9]. In average, the yield of ethanol extract was 12.0% (w/w) from ASMq. ASMq ethanol extract used in the experiments was dissolved in distilled water as a 100 mg/mL (w/v) stock solution and sterilized by 0.45 *μ*m Millipore filter unit for experimental use. The stock solution was further diluted with cell culture medium (DMEM) for cell culture and treatment.

### 2.3. Cell Culture and Treatment

Caco-2 cells, a human colon cancer cell line, were obtained from the American Type Culture Collection (ATCC) and were grown in a high glucose concentration (4.5 g/L) DMEM medium supplemented with 10% FCS, 1% L-glutamine (200 mM), and 1% penicillin-streptomycin (100 IU–100 *μ*g/mL) in a humidified atmosphere of 5% CO_2_-95% air mixture at 37°C. All data presented in this report were obtained at least from three independent experiments.

### 2.4. Cell Viability Assay

The viability of the cells was assessed by MTT (3,4,5-dimethylthiazol-2-yl)-2-5-diphenyltetrazolium bromide) assay, which is based on the reduction of MTT by the mitochondrial dehydrogenase of intact cells to a purple formazan product [10]. Cells (2.5 × 10^4^) were plated in a 96-well plate (Coastar from Corning, NY) and routinely incubated for 24 h at 37°C prior to use. After 24 h they were treated with different concentrations (0.5–7.5 mg/mL) of ASMq ethanol extract for different time intervals (48 and 72 h). After the treatment, media containing ASMq ethanol extract were carefully removed by aspiration. 100 *μ*L of 0.5 mg/mL MTT in cell culture medium was added to each well and incubated for 2 h. 100 *μ*L of 10% SDS, 0.01 M HCl solution was added to each well to dissolve the formazon crystals formed. The plates were covered with aluminum foil and kept in an incubator for 12 h for dissolution of the formed formazan crystals. Amount of formazan was determined measuring the absorbance at 560 nm using a microplate reader. 

### 2.5. DNA Fragmentation Detection

The cells were cultured (5 × 10^6^ cells per mL) in 25 cm^2^ flasks, total volume 5 mL of medium per flask, in the presence of ASMq ethanol extract (0.5–7.5 mg/mL) for different time intervals (24 and 48 h) at 37°C. Controls were performed at the same time with DMEM. After incubation for 24 h, the cell layer was rinsed twice with 5 mL of PBS. To extract DNA, the cells were lysed by incubation for 5 min with 1 mL of lysis buffer (1% N-Lauroyl sarcosine, 20 mM Tris-HCl pH 8.0, 5 mM EDTA), and the cell lysates were collected and transferred into 15 mL corning tubes. Proteins were digested overnight by incubation with 100 *μ*g/mL proteinase K at 37°C, then 7.5 M ammonium acetate and phenol-tris (pH 8)-chloroform (2 : 1, v/v) were added to the homogenate. After centrifugation for 5 min at 2200 ×g at 10°C, one volume of SEVAG (3 mL) was added to the aqueous supernatant and the mixture was centrifuged for 5 min at 2200 ×g at 10°C. The aqueous supernatant was transferred to Eppendorf tubes and incubated for 5 min at 37°C to eliminate traces of chloroform. RNA was digested by incubation with 12 *μ*g/mL RNase A for 30 min at 37°C, and then one volume of SEVAG was added. After centrifugation for 5 min at 2200 ×g at 10°C, the DNA in the aqueous supernatant was precipitated at −10°C for 4 h with ethanol. The mixture was centrifuged for 45 min at 2200 ×g at 4°C, and the supernatant removed. The pellet was rinsed with 70% ethanol, dried at room temperature for 2 h, and resuspended in 200 *μ*L of TE 20–1 (20 mM Tris-HCl pH 8.0, 1 mM EDTA) for DNA quantification by UV spectrophotometry at 254 nm. Loading buffer was added to 10 *μ*g of DNA for each treatment, and the samples were analysed by electrophoresis on a 1% agarose gel (1.5 h at 80 V/30 mA) with a TBE running buffer (44 mM Tris–HCl, 44 mM boric acid, 50 mM EDTA, pH 8.0) [10].

### 2.6. Cell Cycle Analysis by Flow Cytometry

For cell cycle analysis, 5 × 10^5^ cells seeded in 3 mL total volume in 6-well multidishes were incubated as described above for 48 h. Flow cytometric analyses were conducted using a FACScan (Becton Dickinson, France). At the end of incubation, the cells were rinsed twice with PBS and trypsinized in trypsine-0.02% EDTA mixture. After centrifugation for 10 min at 600 ×g at 4°C, the supernatant was removed, the pellet resuspended in 300 *μ*L of PBS, then 700 *μ*L of cold methanol were added and the mixture kept at −20°C for 30 min. After centrifugation for 5 min at 600 ×g and at 4°C, the pellet was treated with 2 mg/mL RNase A at 37°C during 30 min and stained with 50 *μ*g/mL propidium iodide containing 0.1% Triton X-100 and EDTA 0.02 mg/mL. The percentage of cells in each stage of the cell cycle was determined by counting 10^4^ cells, using *cellquest* software (Becton Dickinson, France) [10].

### 2.7. Gene Expression Studies

Expression of apoptosis-related genes, bcl-2, bax, p21, and p53, was studied using reverse transcriptase-PCR (RT-PCR). 5 × 10^5^ cells seeded in 3 mL total volume in 6-well multidishes were incubated with the presence of ASMq ethanol extract (0.5–7.5 mg/mL) for 48 h at 37°C. The housekeeping genes GAPDH were used as control. At the end of incubation, the cells were rinsed twice with PBS and trypsinized in trypsine-0.02% EDTA mixture. After centrifugation for 5 min at 500 ×g at 4°C, the supernatant was removed, and the pellet was used for RT-PCR studies. Total RNA was isolated using SV Total RNA Isolation System (Promega, France). cDNA was generated by Reverse Transcription System (Promega, France). 10 *μ*L of cDNA product was used for PCR reaction as templates. PCR was carried out using the gene-specific upstream and downstream primers (Table 1). Initial denaturation at 95°C for 3 min was followed by a PCR cycle of denaturation at 94°C for 1 min, annealing at 55°C for 1 min, and extension at 72°C for 2 min. PCR products were separated on a 1.5% agarose gel and stained with ethidium bromide [11].

### 2.8. Statistical Analysis

The data are expressed as mean ± standard deviation (SD) for at least three independent determinations in triplicate or quadruplicated for each experimental point. The statistical differences between treated groups and control groups were determined by Student's *t*-test, and *P* < 0.05 was statistically significant difference.

## 3. Results

### 3.1. Inhibition of Cell Growth

**Figure 1:**
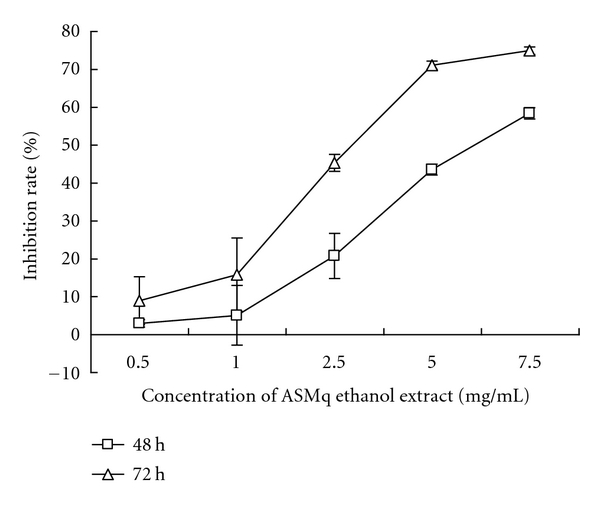
Inhibitory effect of ASMq ethanol extract on Caco-2 cell growth. Cells were incubated with different concentrations of ASMq ethanol extract (0.5–7.5 mg/mL) at 37°C, 5% CO_2_ for 48 or 72 h. The cell growth was determined by the MTT assay. Results are given as mean ± SD from three independent experiments.

Caco-2 cells were used as a model system to examine the effect of ASMq ethanol extract on their growth. The growth inhibitory effect of ASMq ethanol extract was concentration and time dependent (Figure 1). The IC_50_ at 48 and 72 h was 5.99 mg/mL and 3.02 mg/mL, respectively.

### 3.2. Induction of DNA Fragmentation

**Figure 2:**
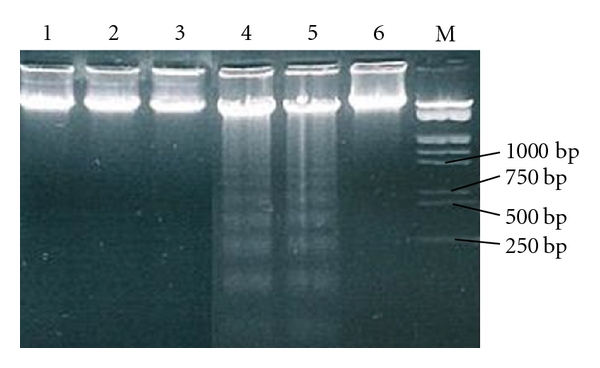
Induction of DNA fragmentation by ASMq ethanol extract in Caco-2 cells. Cells were incubated for 48 h at 37°C with different concentrations of ASMq ethanol extract (0.5–7.5 mg/mL). Lane 1–5: 0.5, 1.0, 2.5, 5.0, and 7.5 mg/mL of ASMq ethanol extract, respectively. Lane 6: cells with no ASMq ethanol extract treatment. Lane M: 250 bp DNA marker.

Results showed that Caco-2 cells treated with ASMq ethanol extract did not induce any DNA fragmentation at the concentration of 0.5–2.5 mg/mL at 48 h of incubation. In contrast, the apoptotic fragments were clearly detected when the cells were treated with a higher concentration of ASMq ethanol extract, 5.0 and 7.5 mg/mL, for 48 h (Figure 2). These observations exhibited that ASMq ethanol extract-induced Caco-2 cells death was possibly mediated through an apoptotic pathway.

### 3.3. Cell Cycle Analysis by Flow Cytometry

**Figure 3:**
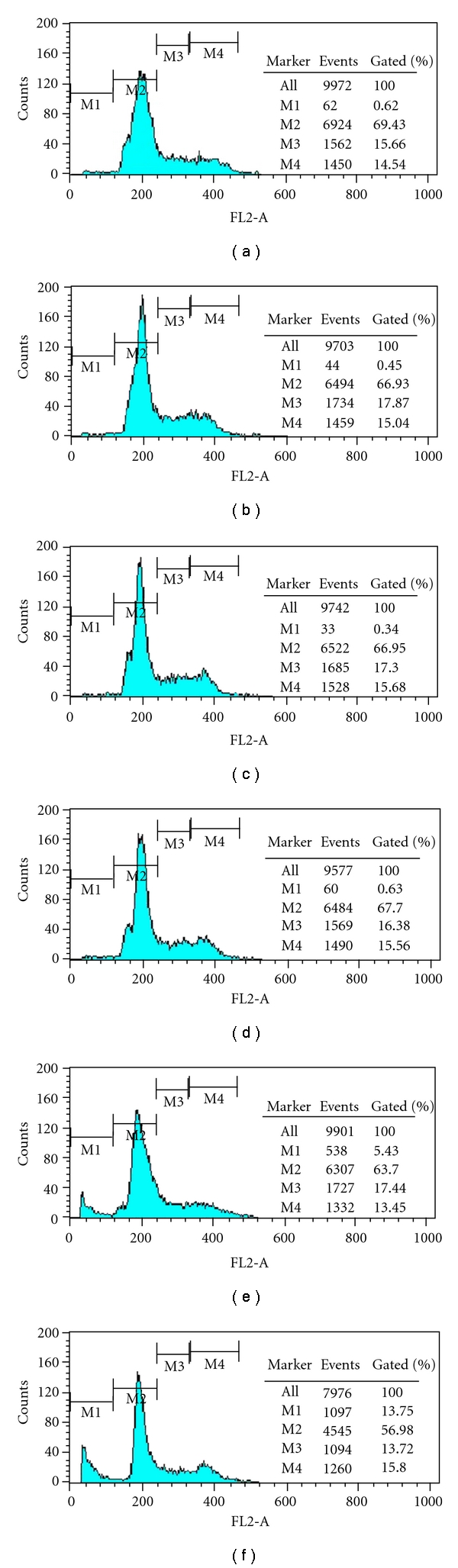
Cell cycle analysis on Caco-2 cells after treatment with ASMq ethanol extract for 48 h. (a) Control without ASMq ethanol extract treatment; (b) Cells treated with 0.5 mg/mL of ASMq ethanol extract; (c) Cells treated with 1.0 mg/mL of ASMq ethanol extract; (d) Cells treated with 2.5 mg/mL of ASMq ethanol extract; (e) Cells treated with 5.0 mg/mL of ASMq ethanol extract; (f) Cells treated with 7.5 mg/mL of ASMq ethanol extract.

Flow cytometry analysis performed on Caco-2 cells after 48 h of incubation with ASMq ethanol extract (0.5–7.5 mg/mL) indicated an alteration in the percentage of cells in each stage of the cell cycle: G0/G1, S and G2/M, as compared to the control (Table 2). We observed at concentrations higher than 5.0 mg/mL an increase in the number of cells in the sub-G1 phase and a decrease in the G0/G1 phase as compared to the control cells (*P* < 0.05). However, the percentage of cells in the S-phase and G2/M phase remained unchanged as compared to the controls (*P* > 0.05). On the other hand, as shown in Figure 3, the sub-G1 peak was detected in a concentration-dependent manner. These results suggested that ASMq ethanol extract had a prominent ability to induce apoptosis in Caco-2 cells. 

### 3.4. Expression of Apoptotic Genes

**Figure 4:**
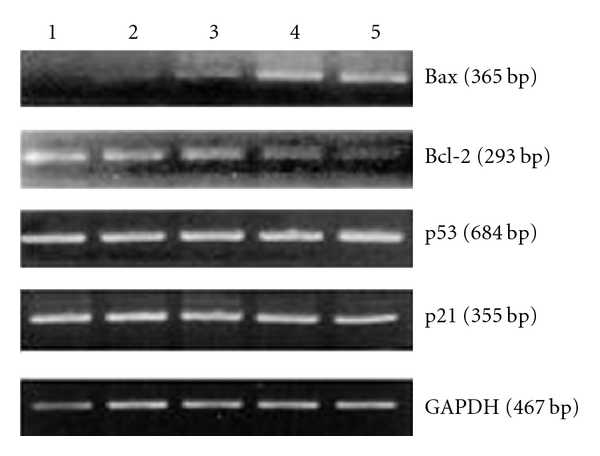
Expression of apoptotic genes in Caco-2 cells treated with ASMq ethanol extracts for 48 h. Column 1, control cells with no treatment; Column 2, cells treated with 1.0 mg/mL of ASMq ethanol extract; Column 3, cells treated with 2.5 mg/mL of ASMq ethanol extract; Column 4, cells treated with 5.0 mg/mL of ASMq ethanol extract; Column 5, cells treated with 7.5 mg/mL of ASMq ethanol extract; Representative data from three individual analyses.

As the results shown in Figure 4, the gene expression of Bax increased and the expression of Bcl-2 decreased concentration dependently in Caco-2 cells after ASMq ethanol extracts treatment for 48 h. But there were not any changes in the expressions of p53 and p21 genes. This indicated that the induction of apoptosis with ASMq ethanol treatment was at least related to the regulation of Bax and Bcl-2 expression, but no relation to the expression of p53 and p21 genes. 

## 4. Discussion

Cancer causes significant morbidity and mortality and is a major public health problem worldwide. An effective cancer prevention program, diet, herb and exercise may decrease the incidence of cancer. Herbs, herbal formulations, and herb-derived compounds are known to have curative potential [3, 6, 12–15]. Abnormal Savda Munziq (ASMq) is a herbal formulation used for cancers in traditional Uighur Medicine and its anticancer activity is well documented [7, 16]. Aqueous extract, ethanol extract, ethyl acetate extract, and total flavonoids of ASMq have been reported to possess inhibitory effect towards a broad range of cancer cells *in vitro* [8, 9, 17–19], and ASMq ethanol extract had chemoprotective effects on DMH-induced colon carcinogenesis [20]. The acting ingredients in ASMq that exerted the anticancer effect may include polyphenols such as flavanoids, which are abundant in ASMq ethanol extract [8]. The molecular mechanism underlying the ASMq ethanol extract induced apoptosis in HepG2 cells has been reported to be associated with increase of caspase-3 activity, DNA fragmentation and Bax/Bcl-2 dependent pathway [9, 19].

Apoptosis, also called the programmed cell death characterized by several morphological and biochemical events, is now known as an important type of cell death in response to cytotoxic treatment. It is a general physiological process to remove unwanted cells without damaging the neighboring cells and inducing inflammatory responses. In recent years, many studies have demonstrated that the dysregulation of apoptosis process is involved in the development of neoplastic transformation and tumor growth. The induction of apoptosis in tumor cells has been shown to be the most common anticancer mechanism conjoint by many cancer therapies. Thus, to find the potential therapeutic antitumor drugs with potent and selective apoptotic effect would be valuable. The administration of many natural compounds with anticancer effect has been shown to be capable of inducing the apoptotic death of cancer cells [21–24]. 

In the present study, we found that ASMq ethanol extract displayed a significant inhibitory effect on the proliferation of Caco-2 cell lines in a dose and time dependently manner. Furthermore, a high-concentration of ASMq ethanol extract (more than 5.0 mg/mL) resulted in significant induction of apoptosis in Caco-2 cells, as evidenced by DNA fragmentation and sub-G1 peak of apoptotic markers detection. More interestingly, the Caco-2 cells treated with ASMq ethanol extract in the concentration of 7.5 mg/mL for 48 h exhibited a dramatic accumulation of cells in sub-G1 phase (13.56%). 

Many genes such as p53, p21, and genes in Bcl-2 family have been demonstrated to play important roles in deciding the initiation and execution of apoptosis in tumor cells exposed to radiation or anticancer drugs. It has been demonstrated that Bcl-2 family members, such as Bcl-2 itself and Bax, are mediators of apoptosis [24]. The balance of proapoptotic Bax and antiapoptotic Bcl-2 is known to be important in determining whether cells die or survive. Bax/Bcl-2 ratio in a cell acts to regulate its own susceptibility to apoptosis [25]. In the present study, to clarify the molecular mechanism of apoptosis mediated by ASMq ethanol extract, we examined the expression of genes including p53, p21, Bax, and Bcl-2 by RT-PCR. Our studies indicated that ASMq ethanol extract-induced apoptosis in Caco-2 cells accompanied by the dose-dependent down-regulation of Bcl-2 gene expression and upregulation of Bax gene expression, while p53 and p21 were not significantly changed. 

The present study suggested that the anticancer effect of ASMq ethanol extract was mediated through multiple pathways. ASMq ethanol extract inhibits cell growth and induces DNA fragmentation and apoptosis in a concentration-dependent manner. Induction of apoptosis is possibly related with Bcl-2 and Bax dependent pathway, but independent of p53 and p21 gene expression. In addition, as a herbal medicine, ASMq ethanol extract has its unique properties of low cost, easy oral consumption, and a long history of use by the Uighur population, all of which are indicative of its potential application as an anticancer agent.

## Figures and Tables

**Table 1:** Oligonucleotides used in the gene expression studies.

GAPDH	sense: 5′-TTCATTGACCTCAACTACAT-3′ antisense: 5′-GAGGGGCCATCCACAGTCTT-3′	467 bp
Bcl-2	sense: 5′-TGCACCTGACGCCCTTCAC -3′ antisense: 5′-AGACAGCCAGGAGAAATCAAACAG-3′	293 bp
p53	sense: 5′-AAACCTACCAGGGCAGCTA -3′ antisense: 5′-ACTGGATGGAGAATATTTCA-3′	684 bp
p21	sense: 5′-CATGTCAGAACCGGCTGGGGATG-3′ antisense: 5′-ACCTGTCACTGTCTTG*T*ACC-3′	355 bp
Bax	sense: 5′-ACCAAGAAGCTGAGCGAGTGTC-3′ antisense: 5′-GGCAGACCGTGACCATCTTTGT-3′	365 bp

**Table 2:** Effect of ASMq ethanol extracts on cell cycle distribution in Caco-2 cells (mean ± SD).

Groups	Percentage of cells in each stage (%)			
	sub-G1 (M1)	G0/G1 (M2)	S (M3)	G2/M (M4)
Control	0.53 ± 0.13	69.79 ± 0.51	14.60 ± 1.50	15.29 ± 1.05
0.5 mg/mL	0.36 ± 0.13	65.35 ± 2.23	18.38 ± 0.71	16.21 ± 1.65
1.0 mg/mL	0.47 ± 0.18	65.70 ± 1.77	16.62 ± 0.97	17.51 ± 2.59
2.5 mg/mL	0.55 ± 0.12	67.12 ± 0.83	16.09 ± 0.42	16.59 ± 1.46
5.0 mg/mL	5.74 ± 1.51*	61.73 ± 0.80*	16.23 ± 0.19	16.09 ± 0.45
7.5 mg/mL	13.56 ± 0.65*	54.69 ± 3.24*	15.81 ± 0.13	16.18 ± 0.53

*P < 0.05, as compared with control culture Representative data from three individual analyses.
